# Multidrug-Resistant *Salmonella enterica* Serotype Typhi, Gulf of Guinea Region, Africa

**DOI:** 10.3201/eid2104.141355

**Published:** 2015-04

**Authors:** Murielle Baltazar, Antoinette Ngandjio, Kathryn Elizabeth Holt, Elodie Lepillet, Maria Pardos de la Gandara, Jean-Marc Collard, Raymond Bercion, Ariane Nzouankeu, Simon Le Hello, Gordon Dougan, Marie-Christine Fonkoua, François-Xavier Weill

**Affiliations:** Institut Pasteur, Paris, France (M. Baltazar, E. Lepillet, M. Pardos de la Gandara, S. Le Hello, F.-X. Weill);; Centre Pasteur du Cameroun, Yaoundé, Cameroon (A. Ngandjio, A. Nzouankeu, M.-C. Fonkoua);; University of Melbourne, Melbourne, Victoria, Australia (K.E. Holt);; Wellcome Trust Sanger Institute, Cambridge, UK (K.E. Holt, G. Dougan);; Scientific Institute of Public Health, Brussels, Belgium (J.-M. Collard);; Institut Pasteur de Bangui, Bangui, Central African Republic (R. Bercion)

## Abstract

We identified 3 lineages among multidrug-resistant (MDR) *Salmonella enterica* serotype Typhi isolates in the Gulf of Guinea region in Africa during the 2000s. However, the MDR H58 haplotype, which predominates in southern Asia and Kenya, was not identified. MDR quinolone-susceptible isolates contained a 190-kb incHI1 pST2 plasmid or a 50-kb incN pST3 plasmid.

Typhoid fever, which is caused by *Salmonella enterica* serotype Typhi, is endemic to the developing world; there were an estimated 26.7 million cases in 2010 (*1*). The incidence of typhoid fever in sub-Saharan Africa was an estimated 725 cases/100,000 persons in 2010, despite a lack of incidence studies conducted in West and central Africa (*1*). Antimicrobial susceptibility data are also scarce for this part of Africa. This issue is problematic because treatment with appropriate antimicrobial drugs is essential for recovery in the context of the global emergence of multidrug resistance.

In the Indian subcontinent and Southeast Asia, the multidrug-resistant (MDR) *Salmonella* Typhi H58 clone, which was named after its haplotype (a combination of defined chromosomal single-nucleotide polymorphisms [SNPs]) (*2**,**3*), has spread rapidly and become endemic and predominant. During the 1990s, this clone acquired a large conjugative incHI1 pST6 plasmid encoding resistance to ampicillin, chloramphenicol, and co-trimoxazole (*4**,**5*); also in the 1990s, this MDR clone became resistant to quinolones and showed decreased susceptibility to ciprofloxacin because of point mutations in the chromosomal *gyrA* gene (*2*). The H58 clone has also spread to eastern Africa, where it has been the most prevalent haplotype (87%) in Kenya since the early 2000s (*6*).

During 1997–2011, high incidence of MDR *Salmonella* Typhi was reported in some countries near the Gulf of Guinea in Africa, including Nigeria (*7*), Ghana (*8**,**9*), Togo (*10*), and the Democratic Republic of the Congo (*11*). During 1999–2003, a British surveillance system reported a prevalence of 19% (49/421) for MDR *Salmonella* Typhi isolates among imported cases of typhoid fever acquired in Africa, particularly in Ghana (*12*). However, nothing is known about the genotypes of these isolates, including whether they belong to the spreading MDR H58 clone.

We report data for the occurrence, genotypes, and characterization of the resistance mechanisms of MDR *Salmonella* Typhi isolates. These isolates were obtained from the French National Reference Center for *Salmonella* (FNRC-Salm), Institut Pasteur (Paris, France), and Centre Pasteur du Cameroun (Yaoundé, Cameroon).

## The Study

Almost all *Salmonella* Typhi strains isolated in France are referred to the FNRC-Salm. Most isolates were obtained from travelers or immigrants, most of whom were infected in Africa and Asia. In Cameroon, the Centre Pasteur du Cameroun collects *Salmonella* Typhi isolates from several hospitals in Yaoundé.

Antimicrobial susceptibility testing was performed according to the guidelines of the antibiogram committee of the French Society for Microbiology (http://www.sfm.asso.fr/nouv/general.php?pa = 2). Isolates were considered to be MDR if they were resistant to ≥2 of the following antimicrobial drugs: amoxicillin, co-trimoxazole (trimethoprim/sulfamethoxazole), chloramphenicol, or tetracyclines.

During 1996–2013, a total of 1,746 *Salmonella* Typhi isolates were collected through the French national surveillance system and subjected to antimicrobial susceptibility testing; 408 were acquired in sub-Saharan Africa (n = 237) and northern Africa (n = 171), and 55 (13.5%) of those acquired in Africa were MDR (Table). All but 1 of the MDR isolates were acquired in sub-Saharan Africa (Table). The proportion of MDR isolates increased from 0% during 1996–1999 to 22.3% (n = 23) during 2010–2013. Only 4 isolates from Africa were resistant to nalidixic acid, including 1 isolate resistant to ciprofloxacin. Because these isolates acquired after 2010 were not MDR isolates, they were not studied further.

**Table:** Characteristics of *Salmonella enterica* serotype Typhi isolates, France and Cameroon, 1996–2013*

Location	1996–1999	2000–2004	2005–2009	2010–2013
France				
No. isolates studied	345	266	627	508
No. isolates acquired in Africa	86	64	155	103
No. (%) MDR†	0	7 (10.9)	25 (16.1)	23 (22.3)
Country of infection for MDR isolates (no.)		Benin (3), Togo (2), Burkina-Faso (1), Cameroon (1)	Cameroon (7), Côte d’Ivoire (4), Burkina-Faso (3), Angola (2), Congo (1), Mali (1), Benin (1), Nigeria (1), Mauritania (1), Togo (1), Central African Republic (1), Guinea (1), not specified (1)	Côte d’Ivoire (7), Guinea (3), Burkina-Faso (3), Cameroon (2), Congo (1), Central African Republic (1), Niger (1), Mali (1), Nigeria (1), Chad (1), Togo (1), Algeria (1)
Yaoundé, Cameroon				
No. isolates studied	ND	61	75	49
No. (%) MDR	ND	29 (47.5)	50 (66.6)	37 (75.5)

*MDR, multidrug resistant; ND, not determined.  †For isolates acquired in Africa.

In Yaoundé, the proportion of MDR isolates was high (45.5 %, 29/61) in the first survey during 2000–2004. However, this proportion increased to 75.5% (37/49) during 2010–2013.

We studied 61 isolates (Technical Appendix). Of these, 46 were MDR: 29 acquired in Africa and detected at FNRC-Salm before 2010; 2 acquired in France during 2009 at an African festive meal (*13*); 12 randomly selected acquired in Yaoundé during 2002–2007; 2 acquired in the Central African Republic, and 1 acquired in Morocco (*2*). The remaining 15 comparison strains (MDR or drug susceptible) that belonged to various haplotypes and were acquired in Africa and Asia during 1958–2009.

Mechanisms of antimicrobial drug resistance were determined as described (*14*). Genetic diversity and phylogenetic relationships were studied by using standardized *Xba*I–pulsed-field gel electrophoresis (PFGE) (http://www.cdc.gov/pulsenet/pathogens/index.html), haplotyping (*5*), and **clustered regularly interspaced short palindromic repeats (**CRISPR) typing (*15*). Haplotyping was based on identification of SNPs at 1,487 defined chromosomal loci, and CRISPR typing was based on detection of 32-bp sequences (spacers) within 1 or both CRISPR regions.

**Figure 1:**
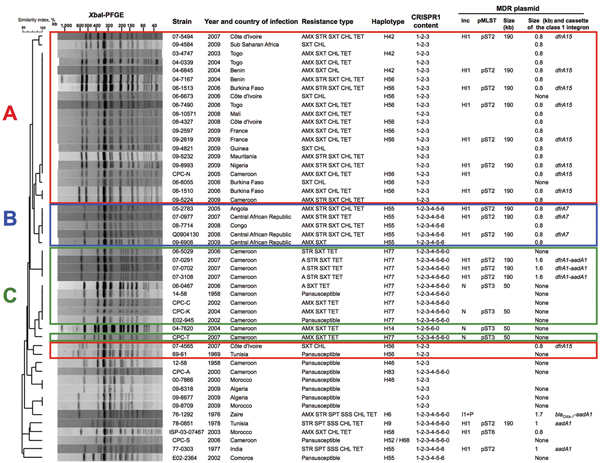
Characteristics of 50 *Salmonella enterica* serotype Typhi isolates. The dendrogram was generated by using BioNumerics version 6.6 software (Applied Maths, Sint-Martens-Latem, Belgium) and shows results of cluster analysis on the basis of *Xba*I–pulsed-field gel electrophoresis (PFGE) fingerprinting. Similarity analysis was performed by using the Dice coefficient, and clustering analysis was performed by using UPGMA. CRISPR1, **clustered regularly interspaced short palindromic repeats 1; MDR, multidrug resistant;** AMX, amoxicillin; STR, streptomycin; SXT, trimethoprim/sulfamethoxazole; CHL, chloramphenicol; TET, tetracycline; SPT, spectinomycin; SSS, sulfamethoxazole. Zaire is the former name of the Democratic Republic of the Congo. Plasmid multilocus sequence typing (pMLST) was performed as described for incN http://pubmlst.org/plasmid/) or incHI1 (*4*). Plasmid size was estimated by using S1 nuclease PFGE (*14*). For isolates from Africa, red indicates linage A, blue indicates lineage B, and green indicates lineage C.

**Figure 2:**
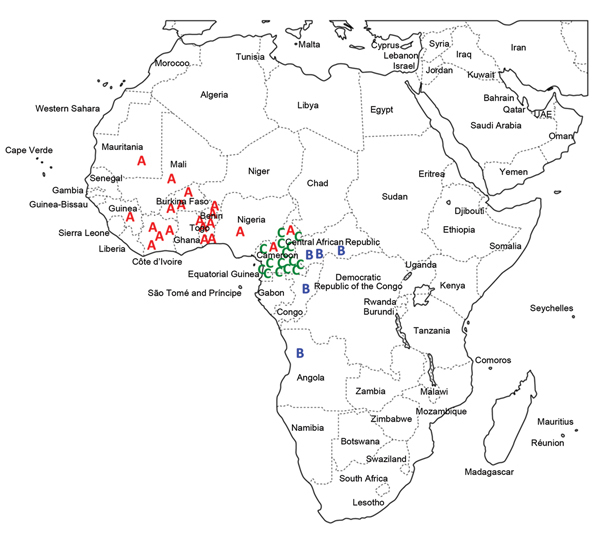
Distribution of multidrug-resistant *Salmonella enterica* serotype Typhi isolates by genetic lineage (A, B, or C), Gulf of Guinea region, Africa. Location within the country of infection/isolation was assigned at random. UAE, United Arab Emirates.

With the exception of the isolate from Morocco (ISP-03-07467) (*2*), none of the isolates had the H58 haplotype or contained the associated incHI1 pST6 MDR plasmid. We found 3 other lineages with different geographic distributions and MDR plasmids (Figures 1, 2).

Lineage A consisted mostly of haplotype H56 isolates and more rarely H42 (which differs from H56 by 2 SNPs) and was found only in the western part of the Gulf of Guinea region. Lineage B consisted of haplotype H55 isolates and was found in the eastern and southern parts of the Gulf of Guinea region. Lineage C consisted of haplotype H77 isolates and was found only in Cameroon. All 3 lineages had distinctive CRISPR1 spacer contents. *Xba*I-PFGE, which used a similarity value of ≥90% as a cutoff, correctly grouped (i.e., concordant with haplotyping and CRISPR results) all but 2 of the MDR isolates from Africa (Figure 1; Technical Appendix).

The 3 lineages contained a large (≈190 kb) conjugative MDR incHI1 pST2 plasmid that differed among lineages. Resistance to trimethoprim was encoded by different class 1 integron gene cassettes: *dfrA15*, *dfrA7*, and *dfrA1* for incHI1 plasmids of lineages A, B, and C, respectively. All incHI1 plasmids from lineage A encoded resistance to chloramphenicol, and none of those from lineage C encoded such resistance. A second smaller (50-kb) MDR plasmid belonging to the incN incompatibility group (pST3 by plasmid multilocus sequence typing), was present mostly in lineage C isolates, but was also found in 1 lineage A isolate (02-1739) (Technical Appendix).

## Conclusions

Analysis of older isolates and previously published data (*2*) showed that susceptible *Salmonella* Typhi strains of haplotypes H42, H56, and H77 had already been identified in Senegal in 1962, Tunisia in 1969, and Cameroon in 1958, respectively. This finding suggests that the MDR isolates from lineages A and C are derived from local *Salmonella* Typhi populations in Africa, rather than being recently imported from other regions to which this bacterium is endemic. Haplotype H55 was previously restricted largely to the Indian subcontinent and eastern Africa (*2*); it was detected in association with an incHI1 pST2 plasmid in India during the mid-1970s (*5*). Therefore, lineage B might have been was imported into central Africa from eastern Africa/southern Asia.

A previous study also reported isolation of an MDR clone in the Democratic Republic of the Congo in 2004 that was resistant to quinolones, showed decreased susceptibility to ciprofloxacin, and belonged to the Asian H58 lineage (*2*). Because only a limited number of isolates from central Africa were tested in our study, studies of a larger collection of isolates might provide more information about bacterial genotypes/MDR plasmids circulating in central Africa.

Despite intrinsic limitations of a laboratory surveillance system for typhoid fever that is used mostly for travelers and immigrants and has the bias of preferential links caused by colonial history and choices of tourist destinations, we documented emergence of 3 MDR *Salmonella* Typhi lineages in the Gulf of Guinea area. Two lineages found in Guinea and Cameroon were local lineages that acquired MDR conjugative plasmids, either a large incHI1 pST2 plasmid or a smaller incN pST3 plasmid. The H58 lineage, which is currently predominant in Asia and eastern Africa, was not detected among MDR isolates from West and central Africa.

**Technical Appendix.** Characteristics of 61 *Salmonella enteric* serotype Typhi isolates and strains studied.

## References

[1] Buckle GC, Walker CL, Black RE. Typhoid fever and paratyphoid fever: systematic review to estimate global morbidity and mortality for 2010. J Glob Health. 2012;2:010401. 10.7189/jogh.01.010401 23198130 PMC3484760

[2] Roumagnac P, Weill FX, Dolecek C, Baker S, Brisse S, Chinh NT, Evolutionary history of *Salmonella* Typhi. Science. 2006;314:1301–4. 10.1126/science.1134933 17124322 PMC2652035

[3] Holt KE, Parkhill J, Mazzoni CJ, Roumagnac P, Weill FX, Goodhead I, High-throughput sequencing provides insights into genome variation and evolution in *Salmonella* Typhi. Nat Genet. 2008;40:987–93. 10.1038/ng.195 18660809 PMC2652037

[4] Phan MD, Kidgell C, Nair S, Holt KE, Turner AK, Hinds J, Variation in *Salmonella enterica* serovar Typhi IncHI1 plasmids during the global spread of resistant typhoid fever. Antimicrob Agents Chemother. 2009;53:716–27. 10.1128/AAC.00645-08 19015365 PMC2630618

[5] Holt KE, Phan MD, Baker S, Duy PT, Nga TV, Nair S, Emergence of a globally dominant IncHI1 plasmid type associated with multiple drug resistant typhoid. PLoS Negl Trop Dis. 2011;5:e1245. 10.1371/journal.pntd.0001245 21811646 PMC3139670

[6] Kariuki S, Revathi G, Kiiru J, Mengo DM, Mwituria J, Muyodi J, Typhoid in Kenya is associated with a dominant multidrug-resistant *Salmonella enterica* serovar Typhi haplotype that is also widespread in Southeast Asia. J Clin Microbiol. 2010;48:2171–6. 10.1128/JCM.01983-09 20392916 PMC2884483

[7] Akinyemi KO, Coker AO. Trends of antibiotic resistance in *Salmonella enterica* serovar typhi isolated from hospitalized patients from 1997 to 2004 in Lagos, Nigeria. Indian J Med Microbiol. 2007;25:436–7. 10.4103/0255-0857.37369 18087113

[8] Mills-Robertson F, Addy ME, Mensah P, Crupper SS. Molecular characterization of antibiotic resistance in clinical *Salmonella typhi* isolated in Ghana. FEMS Microbiol Lett. 2002;215:249–53. 10.1111/j.1574-6968.2002.tb11398.x 12399042

[9] Gross U, Amuzu SK, de Ciman R, Kassimova I, Gross L, Rabsch W, Bacteremia and antimicrobial drug resistance over time, Ghana. Emerg Infect Dis. 2011;17:1879–82. 10.3201/edi1710.110327 22000360 PMC3310671

[10] Dagnra AY, Akolly K, Gbadoe A, Aho K, David M. Emergence of multidrug resistant *Salmonella* strains in Lome (Togo) [in French]. Med Mal Infect. 2007;37:266–9. 10.1016/j.medmal.2007.02.002 17459634

[11] Lunguya O, Lejon V, Phoba MF, Bertrand S, Vanhoof R, Verhaegen J, . *Salmonella* Typhi in the Democratic Republic of the Congo: fluoroquinolone decreased susceptibility on the rise. EPLoS Negl Trop Dis. 2012; 6:e1921. Doe: . Pub 2012 Nov 5. 10.1371/journal.pntd.0001921 PMC3499407 23166855

[12] Cooke FJ, Day M, Wain J, Ward LR, Threlfall EJ. Cases of typhoid fever imported to England, Scotland and Wales (2000–2003). Trans R Soc Trop Med Hyg. 2007;101:398–404. 10.1016/j.trstmh.2006.07.005 17014877

[13] Loury P, Tillaut H, Faisant M, Paillereau N, Marquis M, Mari C, Cluster of typhoid fever cases in Ille-et-Vilaine (France), April 2009 [in French]. Bull Epidémiol Hebd. 2010;44:446–8.

[14] Le Hello S, Harrois D, Bouchrif B, Sontag L, Elhani D, Guibert V, Highly drug-resistant *Salmonella enterica* serotype Kentucky ST198-X1: a microbiological study. Lancet Infect Dis. 2013;13:672–9. 10.1016/S1473-3099(13)70124-5 23721756

[15] Fabre L, Le Hello S, Roux C, Issenhuth-Jeanjean S, Weill FX. CRISPR is an optimal target for the design of specific PCR assays for *Salmonella enterica* serotypes Typhi and Paratyphi A. PLoS Negl Trop Dis. 2014;8:e2671 . 10.1371/journal.pntd.0002671 24498453 PMC3907412

